# Parenteral iron therapy and phosphorus homeostasis: A review

**DOI:** 10.1002/ajh.26100

**Published:** 2021-02-09

**Authors:** Kamyar Kalantar‐Zadeh, Tomas Ganz, Henry Trumbo, Melvin H. Seid, Lawrence T. Goodnough, Michael A. Levine

**Affiliations:** ^1^ Division of Nephrology and Hypertension and Kidney Transplantation University of California Irvine Orange California USA; ^2^ David Geffen School of Medicine University of California, Los Angeles Los Angeles California USA; ^3^ St. Mary Medical Center Langhorne Pennsylvania USA; ^4^ Department of Obstetrics and Gynecology University of Southern California Verdugo Hills Hospital Glendale California USA; ^5^ Department of Pathology and Medicine Stanford University Stanford California USA; ^6^ Center for Bone Health and Division of Endocrinology and Diabetes Children's Hospital of Philadelphia and University of Pennsylvania Perelman School of Medicine Philadelphia Pennsylvania USA

## Abstract

Phosphorus has an essential role in cellular and extracellular metabolism; maintenance of normal phosphorus homeostasis is critical. Phosphorus homeostasis can be affected by diet and certain medications; some intravenous iron formulations can induce renal phosphate excretion and hypophosphatemia, likely through increasing serum concentrations of intact fibroblast growth factor 23. Case studies provide insights into two types of hypophosphatemia: acute symptomatic and chronic hypophosphatemia, while considering the role of pre‐existing conditions and comorbidities, medications, and intravenous iron. This review examines phosphorus homeostasis and hypophosphatemia, with emphasis on effects of iron deficiency and iron replacement using intravenous iron formulations.

## 1 INTRODUCTION

Phosphorus plays a critical biochemical role through its involvement in cellular and extracellular metabolism, as an integral component of nucleic acids, cell membranes, high‐energy compounds (eg, adenosine triphosphate [ATP]) utilized in metabolism, and through regulating the activity of many enzymes. Phosphorus is also an important component of the hydroxyapatite crystal, which provides mechanical strength to mineralized tissues and participates in maintaining the proper pH of extracellular fluids.^1^, ^2^ Hypophosphatemia is a common laboratory abnormality; however, hypophosphatemia is usually an incidental finding, delaying its diagnosis.^3^ It is relevant to interpret serum phosphorus within age‐dependent reference ranges for normal levels. In adults, normal serum phosphorus is defined as a serum phosphorus level of 2.5 mg/dL (0.8 mmol/L) or greater with the upper limit of normal of 4.5 mg/dL (1.45 mmol/L), but normal serum phosphorus levels are considerably higher in children (4.5–6.5 mg/dL [1.45–2.10 mmol/L]) and newborns (4.3–9.3 mg/dL [1.4–3.0 mmol/L]).^4^, ^5^

The prevalence of hypophosphatemia in the general population is difficult to ascertain because hypophosphatemia is usually asymptomatic. Moreover, serum phosphorus is not routinely measured. By contrast, hypophosphatemia has been documented in 2.2%–3.1% of hospitalized patients and 29%–34% of patients in intensive care.^6^ Most cases of hypophosphatemia are the result of excessive renal loss of phosphorus, but in some patients hypophosphatemia is a consequence of inadequate gastrointestinal absorption or an insufficient amount in parenteral nutrition formulations. Some medications have also been associated with hypophosphatemia including specific formulations of intravenous iron used to treat iron deficiency,^3^ the most common cause of anemia.^7^ These iron formulations have been associated with transient hypophosphatemia,^8^, ^9^, ^10^, ^11^, ^12^ which is usually asymptomatic, although some cases of severe or protracted hypophosphatemia have been reported.^13^, ^14^ This review focuses on the effects of iron deficiency and parenteral iron replacement on phosphorus homeostasis.

### 1.1 Physiology of phosphorus homeostasis

Phosphorus is an abundant element with a widespread distribution. Total body phosphorus in a 70 kg man is about 700 to 800 mg, most of which is within bones and teeth in a complex with calcium as hydroxyapatite; about 14% of phosphorus is in soft tissue in the form of phosphate.^1^ Only 1% of phosphorus is in extracellular fluids^1^, ^2^ where it exists as inorganic phosphate (H_2_PO_4_^−^ or HPO_4_^2−^), and is used as a buffer and for regulation of mineralization. Serum inorganic phosphate represents only a very small percentage of total body phosphorus; however, it can be readily measured and provides information about the status of body phosphorus stores.

Within the cell, organic phosphorus is required for several enzymatic processes in glycolysis, ammoniagenesis, as well as in oxidative phosphorylation, generating chemical energy by ATP formation from adenosine diphosphate. It also influences hemoglobin's oxygen‐carrying capacity through its role in regulation of 2,3‐diphosphoglycerate synthesis. Moreover, phosphorus atoms are components of DNA and RNA bases and phospholipids involved in cellular structure and signaling.^2^

Phosphorus metabolism is regulated by a complex mechanism affected by multiple hormones, influenced by diet, and modulated by plasma pH.^2^, ^11^ Serum phosphorus exhibits a circadian rhythm with lowest levels in late morning and highest at 4:00 p.m. and at 4:00 a.m.,^2^, ^15^ an important consideration when making clinical assessments of serum phosphorus levels. Serum phosphorus concentrations are rapidly increased by gastrointestinal absorption of phosphate after meals. Absorption occurs principally in the duodenum and jejunum by an active sodium‐dependent cotransporter and para cellular sodium‐independent transport, and meals containing large amounts of phosphate can increase serum phosphorus levels within an hour of eating. Plasma phosphorus enters extracellular fluid and is transported into cells and the skeleton and is filtered in the kidney, with reabsorption in the renal proximal tubule providing the ultimate control of phosphorus balance.^11^, ^16^, ^17^ About 85%–90% of phosphorus filtered into the renal proximal tubule is reabsorbed by the kidney through the sodium‐phosphate cotransporters NaPi2a and NaPi2c.^11^, ^16^

**FIGURE 1:**
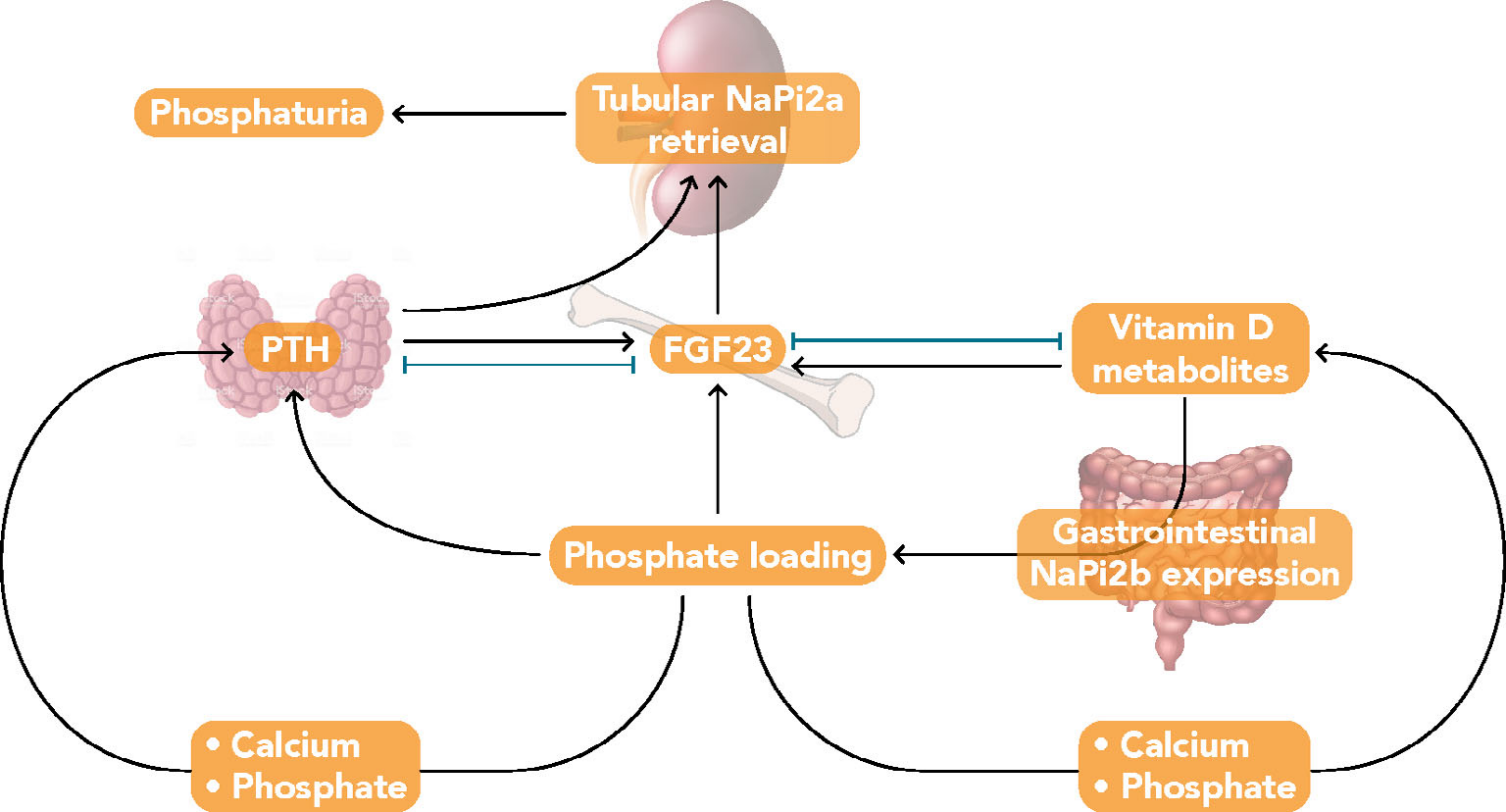
Phosphate homeostasis in healthy individuals.^18^, ^19^ Phosphate loading induces fibroblast growth factor 23 (FGF23) and parathyroid hormone expression, stimulating tubular retrieval of the phosphate channel NaPi‐2a, thus limiting phosphate reabsorption. Two endocrine negative feedback loops control FGF23 expression: one involving parathyroid hormone (left) and the other involving vitamin D (right) [Color figure can be viewed at wileyonlinelibrary.com]

**FIGURE 2:**
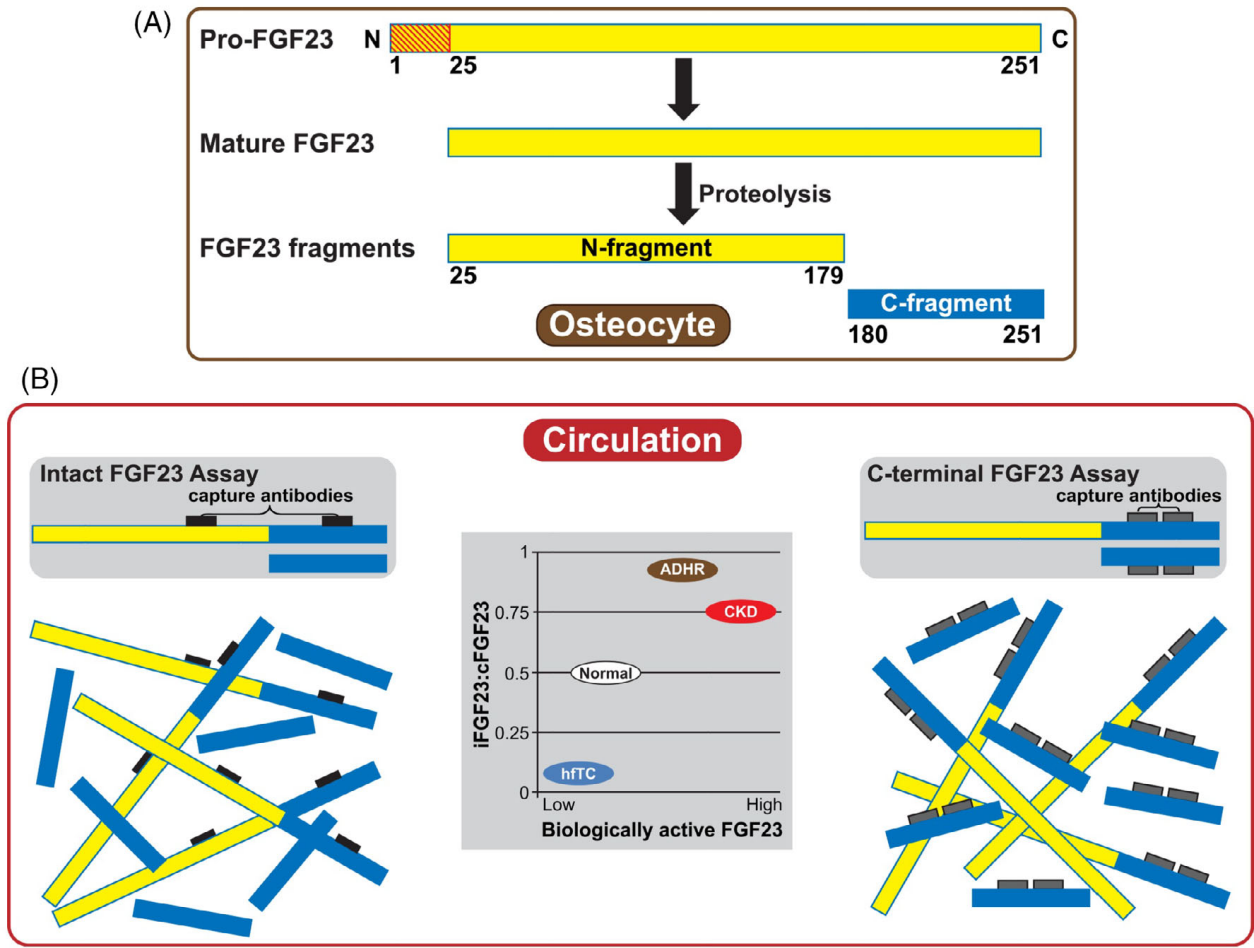
(A) Posttranslational cleavage of FGF23, which occurs primarily in mineralized tissue cells (eg, osteoblasts, odontoblasts and cementoblasts), is an important but not well understood regulatory mechanism that modulates the biological activity of FGF23 during iron deficiency and other pathological conditions.^21^ (B) Assays are available to measure both total and intact FGF23 but they are not standardized or in wide clinical use. Adapted with permission from Wolf M, White KE. Coupling fibroblast growth factor 23 production and cleavage: iron deficiency, rickets, and kidney disease. *Curr Opin Nephrol Hypertens*. 2014;23 (4):411‐419. doi: 10.1097/01.mnh.0000447020.74593.6f ©2014 Lippincott Williams and Wilkins. Abbreviations: ADHR, autosomal dominant hypophosphatemic rickets; cFGF23, C‐terminal region of FGF23; CKD, chronic kidney disease, FGF23, fibroblast growth factor 23; hfTC; hyperphosphatemic familial tumoral calcinosis; iFGF23, intact FGF23 [Color figure can be viewed at wileyonlinelibrary.com]

Fibroblast growth factor 23 (FGF23), parathyroid hormone (PTH), and 1,25‐dihydroxyvitamin D (1,25[OH]_2_D); the biologically active form of vitamin D) are the principal regulators of phosphorus homeostasis (Figure 1).^18^, ^19^ Parathyroid hormone increases renal excretion of phosphate by stimulating endocytosis of NaPi2a and NaPi2c from the apical membrane of proximal tubule cells.^11^, ^20^ Fibroblast growth factor23 regulates serum phosphorus in a negative feedback loop involving the kidney and parathyroid gland. Fibroblast growth factor23 is expressed primarily by mineralized tissue cells (ie, osteoblasts, cementoblasts, and odontoblasts; Figure 2A)^22^ and synthesis is increased in response to dietary load of phosphorus, elevated serum levels of phosphorus,^23^ and 1,25(OH)_2_D,^17^, ^24^ as well as by iron deficiency.^25^ Fibroblast growth factor23 decreases renal phosphate reabsorption by downregulating expression of NaPi2a and NaPi2c,^11^ thereby promoting phosphaturia. Fibrobalst growth factor23 also regulates vitamin D homeostasis^26^ directly by inhibiting transcription of *CYP27B1*, the 25‐hydroxyvitamin D 1α‐hydroxylase, and indirectly through upregulation of *CYP24A1*, the 24‐hydroxylase,^27^ which results in reduced concentrations of 1,25(OH)_2_D^11^ that further decrease serum phosphorus levels.

Most FGF23 action requires binding to a co‐receptor complex composed of an FGF receptor (FGFR) and Klotho, which increases FGFR‐binding affinity of FGF23 by about 20‐fold.^28^ There are four different FGFR isoforms (ie, FGFR1‐4), which are receptor tyrosine kinases. Alternative splicing events can result in different types of FGFR1‐3 isoforms, designated as b‐splice and c‐splice variants, and consequently, can lead to a broad spectrum of FGFRs having altered FGF‐binding specificities.^29^ Additionally, the affinity of these FGFR isoforms for particular FGF ligands differs. In the presence of α‐Klotho, binding of FGF23 to c‐splice variants of FGFR1‐3 and to FGFR4 can occur and result in activation of mitogen‐activated protein kinase signaling.^30^ Fibroblast growth factor receptor1c in concert with α‐Klotho appears to be the primary FGFR in FGF23's physiologic target organs. In contrast to FGFRs, which are ubiquitously expressed, Klotho expression is limited to a few tissues, thus targeting action of FGF23. The Klotho‐FGFR complex is expressed primarily in the kidney (proximal and distal tubules) and parathyroid gland.^30^, ^31^ Some rodent studies have shown binding of this complex on parathyroid cells leads to downstream signaling pathways that decrease secretion of PTH. In the kidney, activation of this complex suppresses 1,25(OH)_2_D synthesis in proximal tubules. Thus, FGF23 appears to have direct and distinct effects through the Klotho‐FGFR complex in proximal and distal renal tubules.^31^ In proximal renal tubule cells, FGF23 binding leads to decreases in membrane expression of NaPi2a and NaPi2c through activation of extracellular signal‐related kinase 1/2 (ERK1/2) and serum/glucocorticoid‐regulated kinase (SGK1) signaling cascades.^31^, ^32^ In distal renal tubules, activation of ERK1/2 and SGK1 signaling pathways stimulates the with‐no‐lysine kinase 1/4 complex, which results in increased membrane expression of calcium and sodium transporters and increased reabsorption of these minerals.^31^

Production of intact FGF23 (iFGF23) involves posttranslational modifications that reduce furin‐mediated peptide cleavage.^17^ By contrast, biologically inactive C‐terminal fragments (cFGF23) are generated by furin cleavage of the intact protein.^33^ Both iFGF23 and inactive cFGF23 fragments are present in the circulation. Because commercially‐available assays utilize antibodies that react with epitopes in the C‐terminal region of FGF23, they detect biologically active iFGF23 as well as inactive cFGF23, which is the predominant immunoreactive form.^21^ By contrast, many investigators use research assays that utilize antibodies which flank the furin cleavage site and react only with iFGF23, but these are not yet approved for clinical use (Figure 2). Under normal conditions, circulating iFGF23 and cFGF23 levels are relatively low, with a mid‐range ratio of iFGF23 to cFGF23 of approximately 0.5.^21^, ^34^ Clinicians should recognize the limitations of these different assays when interpreting circulating concentrations of FGF23. For example, in patients with chronic kidney disease (CKD) and autosomal dominant hypophosphatemic rickets (ADHR), the iFGF23:cFGF23 ratio is ≥0.75. By contrast, patients with hyperphosphatemic familial tumoral calcinosis have very low serum iFGF23 but markedly elevated serum cFGF23.^21^

The available FGF23 assays differ from each other in the epitopes targeted and in reference ranges, and harmonization among tests has not yet been undertaken.^35^ Key considerations for use of FGF23 as a biomarker include proper sample preparation to ensure stability of FGF23 ex vivo; the timing of measurement given the diurnal variation of iFGF23 but only modest change in cFGF23 levels during the day; and accuracy and reproducibility of the assays given limitations in the assays' functional ranges. None of these assays have established reference ranges for age and renal function.^35^

## 2 HYPOPHOSPHATEMIA: PRESENTATION, DIAGNOSIS, CAUSES, AND TREATMENT

### 2.1 Presentation

Signs and symptoms of hypophosphatemia are often nonspecific with generalized weakness being the most common clinical feature. Severe hypophosphatemia may cause neurological problems such as altered mental status, dysarthria, neuropathy, muscle weakness, paresthesia, and seizures.^2^, ^3^, ^18^ Acute, severe hypophosphatemia has been associated with impaired respiratory and cardiac function as well as increased mortality.^2^, ^3^, ^18^ In some patients, muscle pain may accompany weakness, and can indicate hypophosphatemia‐induced rhabdomyolysis.^3^ Hypophosphatemic osteomalacia can occur with chronic hypophosphatemia.

### 2.2 Diagnosis

Assays measuring inorganic phosphate in the blood are used to determine serum phosphorus, which is normally 2.5–4.5 mg/dL (0.80–1.45 mmol/L) in adults.^2^ However, because only a very small proportion of total phosphorus is in circulation, the serum phosphorus concentration may not be a reliable indicator of total body phosphorus content.^2^ Serum phosphorus varies with age, sex, time of day, and food intake^1^, ^18^; thus, it is important to use sex‐related and age‐related reference values, and it is best to collect fasting blood and urine samples in the morning. The serum level of phosphorus exhibits a circadian rhythm, with an estimated 30%–45% fluctuation between the lowest and highest levels over 24 hours (Figure S1).^2^, ^15^

A formal clinical classification of hypophosphatemia in drug trials was often based upon the Common Terminology Criteria for Adverse Events (CTCAE) versions 3^36^ and 4,^37^ which used a grading scale for hypophosphatemia based on actual serum phosphorus levels (Table 1). By contrast, the latest version of CTCAE (v5.0 from November 27, 2017) grades the severity of hypophosphatemia based on clinical severity and necessity of intervention.^38^

**TABLE 1:** National Cancer Institute's Common Terminology Criteria for Adverse Events Versions 3^36^ and 4^37^ compared to Version 5^38^

Terminology Version 3: Metabolic/Laboratory and Version 4: Metabolism and Nutrition Disorders						
Adverse event	Short name	Grade				
		1	2	3	4^a^	5
Phosphate, serum‐low (hypophosphatemia)	Hypophosphatemia	< LLN–2.5 mg/dL < LLN–0.8 mmol/L	< 2.5–2.0 mg/dL < 0.8–0.6 mmol/L	< 2.0–1.0 mg/dL < 0.6–0.3 mmol/L	< 1.0 mg/dL < 0.3 mmol/L	Death

Terminology Version 5 Metabolism and Nutrition Disorders						
CTCAE term		Grade				
		1	2	3	4	5
Hypophosphatemia		Laboratory finding only and intervention not indicated	Oral replacement therapy indicated	Severe or medically significant but not immediately life‐threatening; hospitalization or prolongation of existing hospitalization indicated	Life‐threatening consequences	Death

Abbreviations: CTCAE, Common Terminology Criteria for Adverse Events; LLN, lower limit of normal.

a V 4.0 also included “life‐threatening consequences.”

### 2.3 Causes

Hypophosphatemia is usually caused by acquired or genetic disorders that result in excessive renal phosphate loss.^25^, ^39^ Less commonly, hypophosphatemia is the result of inadequate nutritional intake or impaired absorption of phosphorus (Table 2).^3^ Several methods can be used to assess whether hypophosphatemia is caused by excessive renal loss of phosphorus. Tubular reabsorption of phosphate (TRP) can be calculated from serum and urine concentrations of creatinine and phosphorus, obtained from a 4‐hour to 6‐hour collection of urine in a fasting patient, with a serum sample obtained in the middle of the collection interval. A more accurate assessment of renal phosphate handling can be obtained from calculation of the renal phosphate threshold normalized for the glomerular filtration rate (tubular maximum phosphate reabsorption per glomerular filtration rate, TmP/GFR), which is determined most simply from the Walton and Bijvoet nomogram using both serum phosphorus and TRP; low values indicate renal phosphate wasting.^2^, ^3^, ^18^ It is important to note that the Walton and Bijvoet nomogram may overestimate TmP/GFR in children.^42^ Therefore, in children TmP/GFR is determined as TP/GFR, which specifies tubular phosphate reabsorption under basal conditions. Note, TP/GFR is based on simultaneous measurements of serum and urine phosphorus (SP, UP) and serum and urine creatinine (SCr, UCr; TP/GFR = SP_UP x [SCr/UCr]), which can be determined in both the fasting and non‐fasting child.^42^ The reference range for TmP/GFR is dependent upon both age and gender.

**TABLE 2:** Etiology of hypophosphatemia. (Adapted From Imel and Econs^3^)

Increased renal excretion^2^, ^6^, ^18^, ^34^, ^40^, ^41^	Impaired intestinal absorption/intake^6^	Transcellular shift^2^, ^6^, ^18^
Vitamin D deficiency Acquired or genetic causes of supply, activation, or action Medications Diuretics (hydrochlorothiazide and triamterene) Corticosteroids Bisphosphonates Carbonic anhydrase inhibitors Fanconi syndrome Alcoholism Chronic kidney disease Iron formulations Genetic disorders^25^, ^39^ X‐linked hypophosphatemia Autosomal dominant hypophosphatemic rickets Autosomal recessive hypophosphatemic rickets Hereditary hypophosphatemic rickets with hypercalciuria	Malnutrition Anorexia Low dietary intake of phosphorus Acquired vitamin D deficiency Bariatric surgery Phosphate‐binding medications Sevelamer Antacids containing calcium, magnesium, aluminum Alcoholism	Diabetic ketoacidosis Refeeding syndrome Hyperventilation/respiratory alkalosis Parathyroidectomy/hungry bone syndrome Medications Catecholamines Salicylate poisoning Alcoholism

Vitamin D deficiency is a common cause of acquired hypophosphatemia due to renal phosphate wasting induced by development of secondary hyperparathyroidism (Table 2).^6^, ^18^ In addition, hypophosphatemia can result from a transcellular shift of phosphorus from the extracellular milieu to intracellular stores, which can be severe in cases with underlying phosphorus depletion. Under these conditions, treatment of malnutrition (eg, with glucose‐containing infusions or parenteral nutrition) can lead to “refeeding syndrome,” in which carbohydrate‐stimulated release of insulin leads to an acute shift of phosphorus into cells, potentially resulting in severe and symptomatic hypophosphatemia.^6^, ^18^ The risk of insulin‐induced hypophosphatemia is also increased among patients with poorly controlled diabetes or diabetic ketoacidosis, as these patients exhibit total body phosphorus depletion due to increased loss of renal phosphate associated with hyperglycemia.^6^

Most genetic disorders that cause hypophosphatemia are associated with increased renal phosphate wasting, and are usually associated with increased circulating FGF23 levels.^18^ For example, patients with ADHR have missense mutations in the gene encoding FGF23 which alter the furin recognition site. These mutations prevent proteolytic cleavage of iFGF23, thereby leading to increased circulating levels of iFGF23, the active form of FGF23.^21^, ^33^ By contrast, the mechanism for elevated serum concentrations of FGF23 in X‐linked hypophosphatemia (XLH), the most common form of hypophosphatemic rickets, is less well understood. X‐linked hypophosphatemia is caused by inactivating mutations in the phosphate‐regulating endopeptidase homolog, X‐linked (*PHEX*) gene, but FGF23 is not a substrate for PHEX. Autosomal recessive hypophosphatemia (resulting from mutations in the ectonucleotide pyrophosphatase/phosphodiesterase 1 [*ENPP1*] and dentin matrix protein 1 [*DMP1*] genes), are also associated with increased levels of total FGF23 (measured as cFGF23).^25^, ^39^ Certain neoplasms and somatic genetic syndromes are also associated with excessive FGF23 production, such as hemangiopericytoma and other generally benign mesenchymal tumors, fibrous dysplasia, and cutaneous‐skeletal hypophosphatemia syndrome.^18^

Genetic defects in vitamin D homeostasis lead to secondary hyperparathyroidism, which leads to renal phosphate wasting.^11^, ^18^ Loss of function mutations in the gene encoding the vitamin D receptor cause vitamin D‐dependent rickets type 2A.^40^ Inactivating mutations in genes for hydroxylases mediating production of 1,25(OH)_2_D cause vitamin D‐dependent rickets type 1A (1α‐hydroxylase [*CYP27B1*]) and vitamin D‐dependent rickets type 1B (hepatic 25‐hydroxylase [*CYP2R1*]). An activating mutation in the *CYP3A4* gene enhances oxidization of 1,25(OH)_2_D, leading to vitamin D deficiency (vitamin D‐dependent rickets type 3).^40^

Chronic hypophosphatemia may also arise from causes independent of excess FGF23 or PTH, including primary defects in proximal tubule absorption of phosphorus (Table 2).^40^ Fanconi syndrome (many causes including Dent disease), hereditary hypophosphatemic rickets with hypercalciuria, and other primary disorders of the renal proximal tubule are associated with low or suppressed serum concentrations of FGF23.^25^ It has been shown that mutations in sodium‐phosphate cotransporter genes involved in renal phosphate reabsorption, *SLC34A1* and *SLC34A3*, cause hypophosphatemia in idiopathic infantile hypercalcemia and hereditary hypophosphatemic rickets with hypercalciuria, respectively.^43^, ^44^

Several medication classes are linked to hypophosphatemia (Table 2, Table S1).^3^, ^6^, ^45^ Diuretics, corticosteroids including glucocorticoids, bisphosphonates, and carbonic anhydrase inhibitors can lead to hypophosphatemia by increased renal secretion of phosphate; hypophosphatemia has also been associated with insulin therapy, agents affecting acid–base balance as well as parenteral iron formulations, as will be discussed further. Phosphate‐binding medications can impair intestinal absorption and intake of phosphorus, while catecholamines and salicylate poisoning have been associated with hypophosphatemia following a transcellular shift of phosphate.

Iron deficiency is the most common cause of anemia, with an estimated global prevalence of 33%.^46^ Additionally, iron deficiency anemia accounts for 50% of all anemia cases and affects an estimated 1.24 billion individuals worldwide,^47^ predominantly women and children^7^ and is often seen in combination with other nutritional disorders. Vitamin D affects iron homeostasis and erythropoiesis, and low vitamin D levels have been associated with iron deficiency in both adults and children.^48^, ^49^ Synthesis of FGF23 as well as proteolytic inactivation of iFGF23 is increased in proportion to the severity of iron deficiency in humans.^50^ The increase in FGF23 results from an increase in FGF23 mRNA transcription, which has been demonstrated both in vivo (from a low‐iron diet) and in cultured cells (from treatment with an iron chelator); this may be regulated by the transcription factor hypoxia inducible factor‐1α.^34^ The increase in FGF23 transcription and cleavage is stimulated by not only iron deficiency, but erythropoietin and inflammation as well.^34^ Iron deficiency and inflammation affect an increase in transcription factor hypoxia inducible factor‐1α, which is linked to increasing FGF23 levels with involvement of erythropoietin.^34^ Moreover, FGF23 mRNA is increased in cells cultured under hypoxic conditions.^51^ These studies suggest that bone‐produced hypoxia inducible factor‐1α may represent a novel therapeutic target to reduce FGF23 levels, for example in patients with CKD, to minimize the negative consequences associated with FGF23 excess. Taken in context, there is overlap in responses to iron deficiency and hypoxia, both of which are conditions that ultimately result in reduced oxygen delivery to cells.

Iron deficiency increases synthesis of FGF23 and also increases cleavage of iFGF23 into inactive cFGF23, which typically results in little or no change in the circulating level of iFGF23. Iron replacement actually decreases production of FGF23.^50^, ^52^ However, the conundrum is that certain iron formulations, such as ferric carboxymaltose (FCM), also seem to reduce cleavage of iFGF23. Hence, although total FGF23 production is decreased by FCM, the amount of iFGF23 that is secreted increases, leading to hypophosphatemia.^50^, ^53^ The exact mechanism for reduced cleavage of FGF23 is unknown, but may involve either posttranslational modification of FGF23 to make it less susceptible to cleavage or reduced production of enzymes (ie, furin) that process FGF23.^25^ In ADHR, iron deficiency increases FGF23 synthesis but proteolytic processing is impaired resulting in high iFGF23 levels and consequent hypophosphatemia made worse by iron deficiency.^21^, ^33^

When tolerability and adherence to oral iron becomes an issue, intravenous iron infusion is commonly used as a treatment for iron deficiency anemia (IDA); infusion of some formulations of intravenous iron can increase circulating levels of iFGF23 and result in hypophosphatemia.^54^ Although replacing iron per se appears to reduce production of FGF23, some iron formulations may inhibit proteolytic cleavage of FGF23, thereby increasing circulating levels of iFGF23.

Hypophosphatemia associated with intravenous iron supplementation is reported to be mostly asymptomatic and transient.^9^, ^12^, ^55^, ^56^ One retrospective study showed the median duration of hypophosphatemia to be 41 days following intravenous iron treatment, but that hypophosphatemia in some patients lasted greater than 2 months.^57^ A multivariate model found independent risk factors for incident hypophosphatemia to include abnormal uterine bleeding associated with IDA, higher hemoglobin levels, and lower baseline serum phosphorus levels.^50^ Other possible risk factors include concurrent or prior use of medications affecting proximal renal tubular function, hyperparathyroidism, vitamin D deficiency, a history of gastrointestinal disorders associated with malabsorption of fat‐soluble vitamins or phosphate, and malnutrition. Resolution of most cases of hypophosphatemia occurs within three months.^58^

The use of the following intravenous iron formulations has been associated with the occurrence of hypophosphatemia: ferric derisomaltose (formerly iron isomaltoside),^8^, ^59^, ^60^ FCM,^50^, ^52^, ^56^, ^59^ iron polymaltose,^10^ and iron sucrose.^56^ In clinical trials, the frequencies of a transient decline in serum phosphorus below 2 mg/dL ranged from 5%–20% of patients treated with ferric derisomaltose 1000 mg from the studies in patients with IDA of various etiologies and 1%–2% in studies of patients with CKD.^54^, ^60^, ^61^ Nadir was in the first weeks. In one study of patients with IDA from various causes other than CKD, 3.9% of patients in the ferric derisomaltose 1000 mg group and 2.3% of patients in the iron sucrose 200 mg (≤ 5 injections; 1000 mg cumulative dose recommended) group had hypophosphatemia (serum phosphorus <2 mg/dL).^62^ In two open‐label, randomized clinical studies of patients with IDA without reduced kidney function, the incidence of hypophosphatemia was significantly lower (*p* < 0.001 in both studies) in patients treated with ferric derisomaltose 1000 mg (7.9%–8.1%) vs those treated with FCM 750 mg (73.7%–75.0%).^60^

From the pivotal phase 3 IDA trials of FCM, the pooled incidence of serum phosphorus below 2 mg/dL in FCM‐treated participants was 27%, and 2.1% was reported by study investigators to represent a treatment‐emergent adverse event.^58^ In these studies, hypophosphatemia was not associated with a serious adverse event.^63^, ^64^ Transient hypophosphatemia findings based solely on laboratory measurement of serum phosphorus may have limited clinical significance.^2^, ^54^ By contrast, chronic hypophosphatemia can lead to complications such as osteomalacia, fractures, and rhabdomyolysis. Post‐marketing data for FCM indicates that, in most cases, hypophosphatemia resolved within three months.^58^ Nevertheless, a search of the PubMed database of literature published between January 1, 2008 and May 20, 2020 of randomized controlled trials, case studies, and observational studies, identified 12 post‐marketing reports of symptomatic hypophosphatemia leading to osteomalacia or rickets following FCM treatment for iron deficiency.^65^, ^66^, ^67^, ^68^, ^69^, ^70^, ^71^, ^72^, ^73^, ^74^, ^75^, ^76^ A majority of these reports included the use of multiple FCM courses over a prolonged period of time. Some cases were in patients with regular bleeding episodes and underlying comorbidities, which may have lowered basal phosphorus levels independent of FCM treatment. Chronic hypophosphatemia appears to be associated with inflammatory bowel disease or other causes of chronic gastrointestinal blood loss which may lead to treatment with multiple courses of IV iron (eg, FCM) without management of the underlying primary cause of the anemia; consequently, hypophosphatemia is a distinct possibility through the FGF23 activation pathway (Table 3).^65^, ^66^, ^67^, ^68^, ^69^, ^70^, ^71^, ^72^, ^73^, ^74^, ^75^, ^76^ On the other hand, CKD may be protective against hypophosphatemia, as there is reduced capacity for renal excretion of phosphate. Considering that patients with CKD are deemed to be at decreased risk of developing this adverse outcome, clinical trials investigating the incidence of hypophosphatemia occurring with intravenous iron formulations have excluded patients with an estimated GFR < 65 mL/min/1.73 m^2^.^60^

**TABLE 3:** Characteristics of 12 case studies reporting hypophosphatemic osteomalacia after repeated courses of FCM

	Case report	Patient age (years), sex	Underlying disease	Concomitant medication(s)	FCM administration, duration (dose)	Phosphate level after FCM	FGF23	25(OH)D	1,25(OH)_2_D_3_	PTH	Diagnosis
Underlying inflammatory bowel disease/chronic GI blood loss	Reyes et al. 2017^70^	45, Male	Crohn's disease, partial bowel and ileum resection; subtotal colectomy	Varying doses of mesalazine, prednisolone, infliximab and azathioprine	4 years (every 8 weeks)	0.21–0.80 mmol/L (0.65–2.47 mg/dL)	iFGF23 289 pg/mL	75 nmol/L (30 ng/mL)	19 pmol/L (8 pg/mL)	8.3 pmol/L (78 pg/mL)	Hypophosphatemic rickets, that is, osteomalacia
	Schaefer et al. 2017^72^	45, Male	Crohn's disease	Iron sucrose, methylprednisolone (30 mg), azathioprine (150 mg), mesalazine (1.5 g), infliximab and adalimumab (1 year)	Chronic high‐dose for 3 years (27 g total)	0.46 mmol/L (1.42 mg/dL)	FGF23 (total) 173 pg/mL	Normal (value is NA)	NA	Normal	Hypophosphatemic hyperphosphaturic osteomalacia
	Bartko et al. 2018^65^	42, Male	Crohn's disease	Mesalazine, adalimumab and glucocorticoids (long history of use)	1.5 years of high‐dose FCM (1 g/month)	0.5 mmol/L (1.54 mg/dL)	NA	87 nmol/L (35 ng/mL)	55 pmol/L (23 pg/mL)	2.9 pmol/L (27 pg/mL)	FGF23‐related form of hypophosphatemic osteomalacia
	Klein et al. 2018^68^	57, Male	Crohn's disease	Prednisone	26 monthly infusions (750 mg each) for about 2 years	0.87 mmol/L (2.7 mg/dL)	cFGF23 592 RU/mL	NA	221 pmol/L (92 ng/mL)	19 pmol/L (180 pg/mL)	Hypophosphatemic osteomalacia
	Urbina et al. 2018^75^	38, Male	Crohn's disease	Infliximab (4 years)	8 months of FCM (1 g/month)	0.34 mmol/L (1.05 mg/dL)	FGF23 (total) 226 pg/mL	45 nmol/L (18 ng/mL)	19 pmol/L (8 pg/mL)	5514 pmol/L (52 ng/mL)	FGF23‐mediated osteomalacia
	Schaefer et al. 2017^73^	NA	Chronic gastrointestinal blood loss and IDA	NA	2 years (19 g total)	Reduced but value is NA	iFGF23 elevated but value is NA	NA	NA	NA	Hypophosphatemic osteomalacia
	Tournis et al. 2018^74^	31, Male	Hirschsprung's disease; near total colectomy and excision of distal 1/3 of ileum	NA	5 years of monthly FCM therapy, refractory to oral iron	0.419 mmol/L (1.29 mg/dL)	iFGF23 96 pg/mL	77 nmol/L (31 ng/mL)	58 pmol/L (24 pg/mL)	11.1 pmol/L (104.8 pg/mL)	FGF23‐mediated hypophosphatemia
	Fang et al. 2019^67^	73, Female	Hepatitis B cirrhosis, portal hypertensive gastropathy with gastric antral vascular ectasia and IDA	Spironolactone (50 mg daily), entecavir (0.5 mg daily) and a single infusion of denosumab	2 years (11 g in 1 g infusions)	0.27 mmol/L(0.84 mg/dL)	NA	32 nmol/L (13 ng/mL)	NA	29.8 pmol/L (281 pg/mL)	Severe hypophosphatemia and osteomalacia‐related fractures
	Tozzi et al. 2020^76^	61, Female	Hepatitis C cirrhosis, varices, and hypersplenism leading to thrombocytopenia	NA	Monthly FCM infusions for at least 17 months	0.55–0.58 mmol/L (1.7–1.8) mg/dL)	FGF23 361 RU/mL	77 nmol/L (31 ng/mL)	NA	6.7 pmol/L (63 pg/mL)	Hypophosphatemic osteomalacia
Other underlying disease	Moore et al. 2013^69^	50s, Female	IDA with unexplained urinary iron loss	Other IV iron/FCM for 15 years	IV iron for 15 years and FCM recently	Low level (value is NA)	NA	Normal (value is NA)	Normal (value is NA)	Upper normal range	Hypophosphatemic osteomalacia
	Etchenique et. al 2016^66^	59, Male	Hereditary hemorrhagic telangiectasia	NA	Multiple infusions of FCM	0.323 mmol/L (1 mg/dL)	NA	68 nmol/L (27 ng/mL)	NA	3.0 pmol/L (28 pg/mL)	Hypophosphatemic osteomalacia
	Sangrós Sahún et al. 2016^71^	43, Female	IDA due to hypermenorrhea secondary to uterine myomas	NA	5 doses of FCM (100 mg/month)	0.29 mmol/L (0.9 mg/dL)	NA	NA	NA	Elevated but value is NA	Hypophosphatemic osteomalacia

Abbreviations: cFGF23, C‐terminal region of FGF23; FCM, ferric carboxymaltose; FGF23, fibroblast growth factor 23; GI, gastrointestinal; iFGF23, intact FGF23; IDA, iron deficiency anemia; IV, intravenous; NA, not available; PTH, parathyroid hormone; RU, relative units.

### 2.4 Treatment

The appropriate regimen for phosphorus replacement is based upon clinical symptoms, as there are no standardized protocols for management of hypophosphatemia. Mild, asymptomatic cases can be managed with clinical symptom monitoring and watchful waiting. In many cases, patients can receive dietary phosphate or oral phosphate therapy depending on the severity of the deficiency.^18^ Intravenous phosphate is indicated for severe, symptomatic cases, depending on serum phosphorus levels and with chronic hypophosphatemia, supplements may also be administered.^18^ Replacement regimens must consider that serum phosphorus may not be an accurate reflection of the total body phosphorus deficit.^18^

### 2.5 FCM, hypophosphatemia and approach to management

Existing case studies provide insight into the two types of clinical manifestations that have been described herein: acute symptomatic hypophosphatemia which may occur after a single administration of FCM and resolve within weeks, and in rare, isolated instances, chronic hypophosphatemia with bone mineral disorder that may progress to pathological fractures seen after repeated courses of intravenous iron including FCM. The stacking effect of multiple courses of treatment might be prevented by checking serum phosphorus and ensuring normal vitamin D status before giving a repeat course.

A genetic study of patients who experience the acute syndrome could be informative and help identify additional predisposing factors beyond those already known.

## 3 CONCLUSIONS

Serum phosphorus should be monitored in patients with symptomatic hypophosphatemia and those at risk of hypophosphatemia. Patients considered at risk for hypophosphatemia include those with a history of gastrointestinal disorders such as inflammatory bowel disease, conditions associated with malabsorption of fat‐soluble vitamins or phosphate, and other causes of chronic gastrointestinal blood loss. Concurrent or prior use of certain classes of medications that affect proximal renal tubular function, hyperparathyroidism, vitamin D deficiency, menorrhagia, and malnutrition are also contributory factors to an increased risk of hypophosphatemia. Treatment with the IV iron formulation FCM is associated with transient hypophosphatemia in some patients based on pre‐existing risk factors and need for repeat dosing. In instances where repeat dosing with FCM therapy is indicated, monitoring phosphorus levels should be sufficient to justify the use of FCM vs other IV iron formulations. Note, FCM therapy is associated with hypophosphatemia in certain groups of patients. It is key to note that the overall benefit risk profile of FCM remains positive given its robust efficacy and overall worldwide safety experience in patients with ID and IDA.

## AUTHOR CONTRIBUTIONS

Kamyar Kalentar‐Zadeh, Tomas Ganz., Henry Trumbo, Melvin H. Seid, Lawrence T. Goodnough, and Michael A. Levine have contributed to the development and review of the manuscript and approved of the final version of the manuscript for submission.

## CONFLICT OF INTEREST

Kamyar Kalentar‐Zadeh is supported by the National Institute on Aging of the National Institutes of Health grant R21‐AG047036 and the National Institute of Diabetes, Digestive and Kidney Disease grants R01‐DK078106, R01‐DK096920, U01‐DK102163, and K24‐DK091419, as well as philanthropic grants from Mr. Harold Simmons and Mr. Louis Chang. Tomas Ganz is a shareholder and scientific advisor of Intrinsic LifeSciences and Silarus Therapeutics, and consultant for Ionis Pharmaceuticals, Protagonist, Vifor Akebia, Global Blood Therapeutics and Sierra Oncology. Henry Trumbo is a consultant for American Regent, and Daiichi Sankyo. Melvin H. Seid is a consultant for AMAG Pharmaceuticals, American Regent, and Daiichi Sankyo; stockholder of Abbott, AbbVie, Bristol‐Myers Squibb, Gilead Sciences, Johnson & Johnson, Merck, and Pfizer; stockholder and director Periodic Products. Lawrence T. Goodnough is a consultant and scientific advisory board member for American Regent. Michael A. Levine is a consultant for American Regent and Inozyme Pharma and receives research funding from Takeda Pharmaceuticals and Ultragenyx.

## Supporting information


**Figure S1** Circadian variation of serum phosphorus levels in normal subjects.^15^ Adapted with permission from Becker GJ, Walker RG, Hewitson TD, Pedagogos E. Phosphate levels—time for a rethink? *Nephrol Dial Transplant*. 2009;24 (8):2321‐2324. doi: 10.1093/ndt/gfp220 ©2009 Oxford University Press.

**Table S1** Medications that can cause hypophosphatemia^44^

## Data Availability

Data sharing was not applicable to this article as no new data were generated or analyzed during the current study
