# Supplementary material for: Parenteral iron therapy and phosphorus homeostasis: A review
Source: Am J Hematol. 2021 Feb 9;96(5):606–16. doi: 10.1002/ajh.26100 (PMC8248123; doi:10.1002/ajh.26100)

**Figure S1.** Circadian variation of serum phosphorus levels in normal subjects.^15^ Adapted with permission from Becker GJ, Walker RG, Hewitson TD, Pedagogos E. Phosphate levels—time for a rethink? *Nephrol Dial Transplant.* 2009;24(8):2321-2324. doi: 10.1093/ndt/gfp220 ©2009 Oxford University Press.


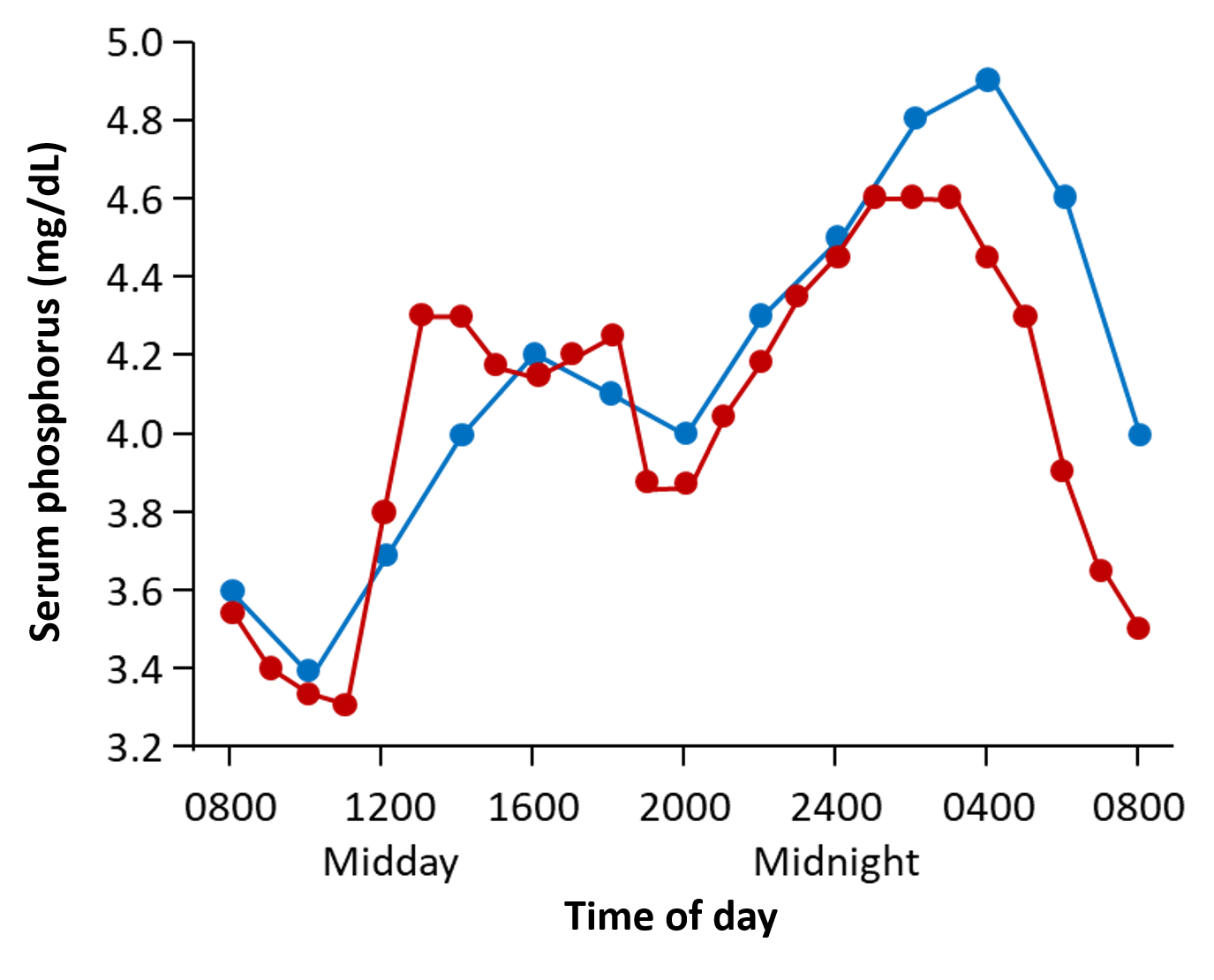

Supplement: Supplementary file 1 — Figure S1 Circadian variation of serum phosphorus levels in normal subjects. 15 Adapted with permission from Becker GJ, Walker RG, Hewitson TD, Pedagogos E. Phosphate levels—time for a rethink? Nephrol Dial Transplant. 2009;24 (8):2321‐2324. doi: 10.1093/ndt/gfp220 ©2009 Oxford University Press. [file AJH-96-606-s001.docx]
