# Supplementary material for: Parenteral iron therapy and phosphorus homeostasis: A review
Source: Am J Hematol. 2021 Feb 9;96(5):606–16. doi: 10.1002/ajh.26100 (PMC8248123; doi:10.1002/ajh.26100)
Supplement: Supplementary file 2 — Table S1 Medications that can cause hypophosphatemia 44 [file AJH-96-606-s002.docx]

**Supplement**

**Table S1.** Medications that can cause hypophosphatemia^44^

| **Drug class** | **Medication** |
| --- | --- |
| Analgesic/antipyretic | Acetaminophen |
| Antiandrogen | Abiraterone acetate |
| Antibiotic | Cefepime, ceftolozane and tazobactam |
| Anticonvulsant | Phenytoin, topiramate |
| Antidepressant | Olanzapine and fluoxetine |
| Antihyperlipidemic | Niacin |
| Antineoplastic | Cabozantinib, carfilzomib, ceritinib, cobimetinib, crizotinib, dabrafenib, dinutuximab, everolimus, imatinib, irinotecan, melphalan, portrazza, nilotinib, panobinostat, pazopanib, rigorafenib, rituximab, romidepsin, sorafenib, tacrolimus, temsirolimus |
| Antiviral | Adefovir dipivoxil, efavirenz/emtricitabine/tenofovir disoproxil fumarate, elvitegravir/cobicistat/emtricitabine/tenofovir disoproxil fumarate, emtricitabine/rilpivirine/tenofovir disoproxil fumarate, emtricitabine /tenofovir disoproxil fumarate, eribulin, foscarnet, tenofovir alafenamide fumarate, tenofovir alafenamide fumarate/emtricitabine, tenofovir alafenamide fumarate/emtricitabine/bictegravir, tenofovir alafenamide fumarate/elvitegravir/cobicistat/emtricitabine, tenofovir alafenamide fumarate/emtricitabine/rilpivarene, tenofovir disoproxil fumarate, tenofovir disoproxil fumarate/emtricitabine/rilpivarene, tenofovir disoproxil fumarate and lamuvidine, tenofovir disoproxil fumarate/efavirenz/lamivudine |
| β-adrenergic blocker | Bisprolol and hydrochlorothiazide |
| Bisphosphonate | Alendronate, pamidronate disodium, risedronate sodium, zoledronic acid |
| CNS agent | Teriflunomide |
| Diuretic | Hydrochlorothiazide, triamterene |
| Hyperammonemia agent | Sodium phenylbutyrate |
| Immunomodulatory agent | Basiliximab, denosumab, lenalidomide, mycophenolic acid, sirolimus, tacrolimus |
| Phosphate binder | Lanthanum carbonate |

CNS, central nervous system
